# Screening and functional analysis of a differential protein profile of human breast cancer

**DOI:** 10.3892/ol.2014.1978

**Published:** 2014-03-18

**Authors:** FU-JUN LIU, XUE-BO WANG, AI-GUO CAO

**Affiliations:** 1 Central Laboratory, Yu-Huang-Ding Hospital, Qingdao University, Yantai, Shandong 264000, P.R. China; 2 Traditional Chinese Medicine Hospital of Jining City, Jining, Shandong 272000, P.R. China

**Keywords:** breast cancer, proteome, immunohistochemistry, bioinformatics, biomarker

## Abstract

To improve the understanding of the enriched functions of proteins and to identify potential biomarkers in human breast cancer, the present study constructed a differentially expressed protein profile by screening immunohistochemistry maps of human breast cancer proteins. A total of 1,688 proteins were found to be differentially expressed in human breast cancer, including 773 upregulated and 915 downregulated proteins. Of these proteins, secreted and membrane proteins were screened and clustered, and more enriched biological functions and pathways were presented in the upregulated protein profiles. Furthermore, altered serum levels of peroxiredoxin (PRDX)2, PRDX6, cathepsin (CTS)B and CTSD were detected by ELISA assay. The present study provides a novel global mapping of potential breast cancer biomarkers that could be used as background to identify the altered pathways in human breast cancer, as well as potential cancer targets.

## Introduction

Breast cancer is one of the most common types of cancer among females worldwide, as well as a leading cause of mortality. However, the survival of patients has increased over the past decades due to earlier diagnosis and effective therapies (1). In addition, cancer biomarkers provide useful information for the prognosis and assessment of cancer treatment.

The identification of cancer biomarkers is important for cancer biology and clinical applications. With the development and improvement of high-throughput biotechnologies, cancer biomarkers can be identified by comparing normal cells with cancer cells through genomic, transcriptomic and proteomic analyses. At present, the most promising biomarkers are proteins (2,3) and proteomic analysis provides an opportunity to identify altered protein groups and the complex pathways in breast cancer. The mapping of proteomic profiles and differential proteomics has been widely performed in breast cancer to identify potential biomarkers (4). Some of these proteins have been reported to have potential clinical significance and key proteins, such as the receptor tyrosine-protein kinase erbB-2 (ERBB2) and breast cancer 1 and 2 early onset, may be used as potential diagnostic, prognostic or predictive biomarkers (5,6). Although cancer markers may indicate the status of cancer development, they alone are not sufficient to determine the cancer biology. In addition, due to the heterogeneity of experimental methods and specimen preparation (7), the proteomic results lack good reproducibility and require further validation prior to their use in clinical detection and to explain the underlying mechanisms of breast cancer. The transformation of normal cells to cancer cells requires the complex regulation of networks and altered molecules. In addition, the networks associated with cancer cause abnormal cell proliferation and invasion. The identification of these intricate pathways is essential to understanding the biological mechanisms of cancer and may aid in predicting or monitoring cancer progression, as well in developing a therapeutic strategy by focusing on the pathways instead of individual proteins. The enriched pathways or functions are the most probable causes of cancer (8,9), and the enriched proteins involved in these processes may in turn serve as target agents in the diagnosis or treatment of cancer.

The aim of cancer proteomics is to identify altered proteins and to correlate them with the tumorigenesis and progression of cancer. The development and application of proteomic technologies has resulted in a surplus of potential breast cancer biomarkers; however, these results require validation by immunohistochemistry or western blot analysis for clinical diagnostics. Immunohistochemistry is being increasingly used in the pathology of breast cancer to provide a definitive histological diagnosis and information for treatment and prognosis. A panel of immunohistochemical markers can be used for estimating prognosis and predicting therapy response (10). Conversely, immunohistochemistry may also be useful for identifying additional cancer markers. Currently, the Human Protein Atlas (www.proteinatlas.org) is used to generate a global immunohistochemistry map of protein expression profiles in normal and cancer tissues, and it provides a reliable resource for the identification of biomarkers. The present study performed a direct comparison of the protein expression levels in breast cancer with those in normal breast tissues to identify differentially expressed proteins in breast cancer. In addition, a functional enrichment analysis was performed to identify new functional modules in breast cancer. The results identified additional potential marker proteins that could be used as background to reveal the altered pathways in human breast cancer. The combinational protein profiles are likely to present a more sensitive and specific evaluation of the heterogeneity of cancer and could be applied to investigate the mechanisms of cancer formation at the functional pathway level.

## Materials and methods

### Patient characteristics and serum collection

Blood samples were collected from 30 breast cancer patients and 30 healthy volunteers at the Yu-Huang-Ding Hospital (Yantai, China) who had provided written informed consent. Ethical approval for the study was obtained from the Yu-Huang-Ding Hospital research and ethics committee. Venous blood was drawn from each subject into 10-ml fasting blood tubes, which were allowed to clot at room temperature for 1 h. The serum was then separated by centrifugation at 2,000 × g for 15 min at 4°C.

### Data collection

Staining profiles for proteins in normal breast and breast cancer tissues were downloaded from the Human Protein Atlas. The expression level of each protein was then graded into four levels: Strong, >75%; moderate, 25–75%; weak, <25% and negative, 0% for use as retrieval parameters. The differentially expressed proteins were defined as those which exhibited a change in expression of more than two levels between the previously described groups. Finally, the selected proteins were grouped into upregulated and downregulated proteins in human breast cancer.

### Broad functional analysis

All differentially expressed proteins were classified broadly into several catalogs according to the Gene Ontology (GO) annotation (www.geneontology.org), PANTHER classification (www.pantherdb.org) and functions annotated in UniProt (www.uniprot.org).

### Over-representation analysis

#### Ontological analysis

The over-representation analyses of GO terms, including biological process and molecular function, were performed using the ConsensusPathDB-human database system (http://cpdb.molgen.mpg.de/CPDB), which is a molecular functional interaction database. The GO level 2 and 3 categories and a P-value cut-off of 0.01 were selected.

### Pathway analysis

The enriched pathway analysis was performed using the DAVID (http://david.abcc.ncifcrf.gov/) and PANTHER tools. For the pathway analysis, the Kyoto Encyclopedia of Genes and Genomes (KEGG) and Reactome databases were selected. The minimum overlap with the input list was set at two proteins, with P<0.01.

### Analysis of the membrane organization

The secreted and membrane proteins were screened through tools in LOCATE (http://locate.imb.uq.edu.au/), which is a curated database for describing the membrane organization. The membrane proteins included type I, II and III proteins.

### ELISA assay

The serum samples were collected from breast cancer patients and healthy age-matched volunteers. The ELISA kits for peroxiredoxin (PRDX)2, PRDX6, cathepsin (CTS)B and CTSD (Abnova Corporation, Taibei, Taiwan) were used and the assays were run according to the manufacturer’s instructions. The absorbance was measured at 450 nm using a 680 microplate reader (Bio-Rad, Hercules, CA, USA).

### Statistical analysis

The ELISA data were statistically analyzed and the differences between the two groups were assessed by the independent samples t-test. P<0.01 was considered to indicate a statistically significant difference.

## Results

### Differentially expressed proteins in human breast cancer

The human breast cancer protein profile was constructed by screening the Human Protein Atlas quantitative dataset stained using immunohistochemistry. A total of 1,688 proteins were found to be differentially expressed between breast cancer and normal breast tissues, including 773 upregulated and 915 downregulated proteins in human breast cancer.

### Broad functional analysis

**Figure 1:**
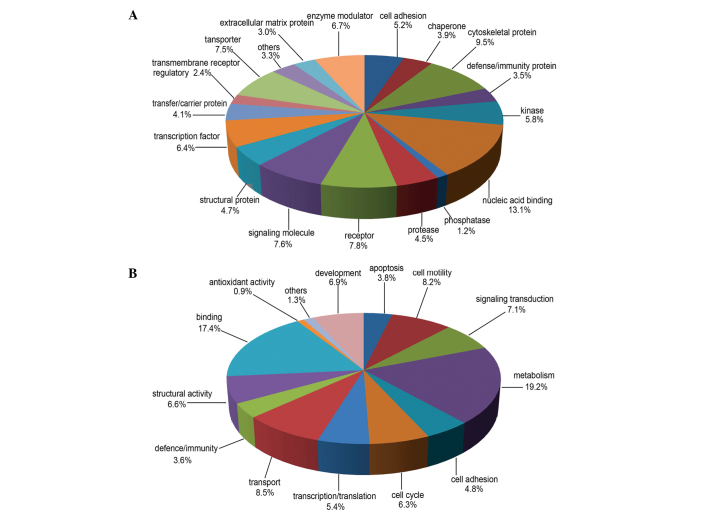
Pie chart of the broad biological functions associated with differentially expressed breast cancer proteins. (A) Protein classes were grouped by the protein classification tool in PANTHER and (B) major functions were categorized using Gene Ontology and PANTHER.

All the proteins were placed into broad functional categories on the basis of the GO and PANTHER databases. As shown in Fig. 1, a total of 1,688 proteins were grouped into several classes according to their major functions. The major protein class was the nucleic acid binding proteins (13.1%), followed by the cytoskeletal proteins (9.5%), receptors (7.8%), signaling molecules (7.6%) and transporters (7.5%). These molecules exhibited different functions, of which the leading function was metabolism (19.2%), followed by binding (17.4%), transport (8.5%) and cell motility (8.2%).

### Enriched ontological analysis

**Figure 2:**
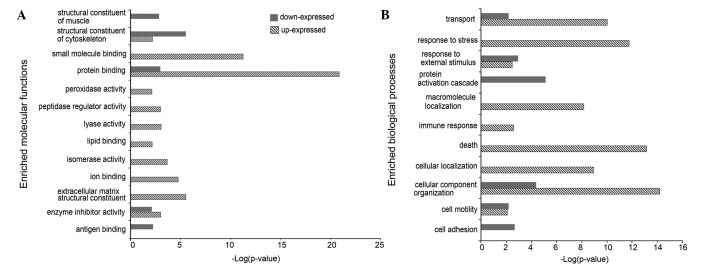
Enrichment analysis of the molecular functions and biological processes of differentially expressed breast cancer proteins. Enriched classification was determined using Gene Ontology. The enrichment P-value of each term was transformed to a −log (P-value).

The main enriched GO terms were categorized with respect to upregulated and downregulated proteins, to determine which molecular functions or biological processes were enriched in the protein groups differentially expressed in human breast cancer (Fig. 2). As to be expected, more important molecular functions and biological processes were enriched with upregulated proteins as opposed to downregulated proteins. The important enriched molecular functions were enzyme activity and binding functions (including peroxidase, lyase, isomerase and peptidase regulator activity), as well as small molecule, lipid and ion binding functions. These enriched molecular functions were identified to be important in the processes of response to stress, cell death and localization.

### Enriched pathway analysis

An enriched pathway analysis of upregulated and downregulated proteins in breast cancer was performed using the KEGG and Reactome pathway databases; in total, 42 pathways were obtained. As predicted, the more important pathways were enriched with upregulated proteins than downregulated proteins. As listed in Table I, 25 pathways were uniquely enriched with upregulated proteins and four pathways were uniquely enriched with downregulated proteins.

### Characteristics of potential cancer markers

The secreted and membrane proteins in cancer tissues are potential markers that may be detected in blood with altered expression. The membrane organization analysis showed that 137 (17.7%) of the 773 upregulated proteins were secreted proteins, with 242 (31.3%) of the 773 upregulated proteins identified as membrane proteins (including 33 type I, 94 type II and 115 type III). In the downregulated protein profiles, 126 (13.7%) proteins were identified to be secreted proteins and 338 (36.9%) proteins were identified to be membrane proteins (including 50 type I, 122 type II and 166 type III) (Table II).

### Validation of selected secreted proteins

**Figure 3:**
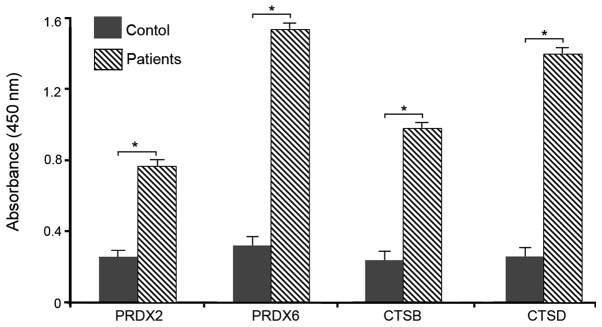
ELISA analysis of serum PRDX2, PRDX6, CTSB and CTSD levels in the breast cancer patients and healthy volunteers. ^*^P<0.01. PRDX, peroxiredoxin; CTS, cathepsin.

ELISA was performed to confirm the altered expression levels of PRDX2, PRDX6, CTSB and CTSD in the serum among the breast cancer patients and healthy volunteers as a representative sample in order to validate the serum levels. The results indicated that the four proteins were upregulated in the serum of breast cancer patients (Fig. 3).

## Discussion

Breast cancer is one of the most common types of cancer among females caused by the accumulation of gene mutations combined with altered gene regulation and protein pathways. Therefore, the identification of markers to predict, diagnose and treat breast cancer is of critical importance. By constructing protein profiles associated with breast cancer, proteins with altered expression can be identified to further investigate and decipher the complex signaling networks involved in tumorigenicity and cancer progression. In the present study, a differentially expressed protein profile associated with human breast cancer was constructed by quantitatively comparing the credible immunohistochemistry results of breast cancer tissues with those of normal breast tissues. The results provided a novel global analysis of human breast cancer markers.

As predicted, certain well-known breast cancer markers, such as the anterior gradient homolog 2 (11), ERBB2 (12) and Rho-associated protein kinase-2 (13) which are the overexpressed in breast cancer, were included in the present study. Based on the GO and PANTHER analyses, the differentially expressed proteins in human breast cancer were grouped according to their major biological functions. The functional category is useful for investigating the mechanisms of breast cancer formation and progression. The leading function was metabolism (19.2% of proteins), which is an emerging hallmark of cancer (14). The following functions, such as binding, transportation, signaling transduction and cell cycle functions, are known to be associated with tumorigenesis (15). In addition, the functions of cell motility and adhesion are involved in the progression of cancer invasion and metastasis (16,17).

The enriched functional terms identified in the upregulated breast cancer proteins may account for cancer development and progression. In addition, several functional terms are considered to be involved in breast cancer, for example, PRDX is reportedly overexpressed in breast cancer (18) and may be used as a marker for breast cancer (19). In the present study, four PRDX members (PRDX1, 2, 4 and 6) were identified to exhibit upregulated levels of expression in the breast cancer tissues. Peroxidase enzymes are considered to be important in eliminating the peroxides generated during cancer metabolism, and PRDX1 and 2 in MCF-7 breast cancer cells exhibit important functions as inhibitors of cell death during the cellular response to oxidative stress (20). In addition, the overexpression of PRDX6 leads to a more invasive phenotype and metastatic potential of human breast cancer (21). An additional protein family of interest is the CTS proteins; in the present study, five CTS proteins (CTSB, CTSC, CTSD, CTSH and CTSZ) were identified to be upregulated in breast cancer. CTSs are overexpressed in breast cancer and, as a result, have been suggested to be biological markers for prognosis (22). Furthermore, CTSB and CTSH have been reported to be overexpressed in inflammatory breast cancer, as well as involved in cancer progression and invasion (23). The downregulated proteins in human breast cancer predominantly belong to the structural constituents of the muscle and cytoskeleton, as well as small molecule or antigen binding. These proteins are significantly involved in transport, protein activation and cell adhesion. In addition, the low expression levels of specific proteins have also been associated with cancer progression, such as the signal transducer and activator of transcription-5a, which showed reduced expression in primary breast cancer and is subsequently an independent marker of poor prognosis (24).

As predicted, in the present study, several well-known cancer pathways, such as glycolysis, the cell cycle and the phosphoinositide 3-kinase-Akt signaling pathway (25), were identified and confirmed the reliability of the pathway analyses used. The most enriched pathway with upregulated proteins was glycolysis, which indicated that breast cancer relies on the production of ATP by glycolysis for proliferation and progression. The overall activities of the pathways determine the invasive and metastatic phenotype of cancer cells. Thus, pathological analysis of the constituents of the pathway and development of the inhibitors directed at the pathway are likely to have clinical benefits in the diagnosis, prognosis and treatment of breast cancer. These complex pathways and networks are highly regulated and the alteration of specific molecules may lead to the development of cancer.

The membrane proteins are considered to be potentially effective therapeutic targets and the secreted proteins may serve as biomarkers for cancer (26). Thus, these proteins were screened using the Membrane Organization tool (LOCATE) and were found to exhibit different localization characteristics, including intracellular proteins (that executed the previously described functions) and extracellular proteins, which may be useful biomarkers. A total of 137 secreted proteins and 242 membrane proteins were found to be upregulated in human breast cancer. Generally, cancer cells decrease the number of cell-cell interactions and increase the number of cell-extracellular matrix interactions, which subsequently results in cancer metastasis. Therefore, proteins secreted from cancer cells, such as CTS and PRDX, may serve as promising biomarkers of cancer cell migration, invasion and angiogenesis. Analyzing the expression of these proteins in blood specimens may aid in the determination of a diagnosis of breast cancer, as a molecular diagnostic tool. Certain secreted and membrane proteins serve as signals for cell communication and control cell proliferation, differentiation and other physiological functions. For example, the secreted proteins conjugative transfer region 1 and stanniocalcin 2 serve as potential prognostic markers in breast cancer (27).

In conclusion, the present study constructed and characterized a novel protein profile associated with human breast cancer. However, further studies are warranted to confirm the enriched functions and pathways. These results may be used as a reliable resource to identify the altered pathways in human breast cancer, as well as potential cancer targets for the early diagnosis, therapeutic targets and disease response markers of breast cancer.

## Figures and Tables

**Table I:** Enriched pathways in upregulated and downregulated breast cancer proteins.

		Upregulated		Downregulated		
				
Pathway name	Set size	n (%)	P-value	n (%)	P-value	Data source
Glycolysis	29	11 (40.7)	6.82E-08	-	-	Reactome
Glucose metabolism	67	16 (24.6)	2.68E-07	-	-	Reactome
Proteasome	44	13 (29.5)	3.61E-07	-	-	KEGG
Folding of actin by CCT/TriC	9	6 (66.7)	1.88E-06	-	-	Reactome
Focal adhesion	206	26 (12.7)	4.96E-05	24 (11.7)	1.29E-04	KEGG
SHC-mediated signaling	15	6 (40.0)	8.44E-05	-	-	Reactome
RAF/MAP kinase cascade	10	5 (50.0)	9.54E-05	-	-	Reactome
Citrate cycle (TCA cycle)	30	8 (26.7)	1.51E-04	7 (23.3)	6.49E-04	KEGG
Calnexin/calreticulin cycle	11	5 (45.5)	1.67E-04	-	-	Reactome
Smooth muscle contraction	24	7 (29.2)	2.13E-04	6 (25.0)	1.08E-03	Reactome
Metabolism of proteins	572	48 (9.1)	3.04E-04	-	-	Reactome
Mitotic prophase	35	8 (23.5)	3.85E-04	-	-	Reactome
Membrane trafficking	154	19 (12.7)	5.10E-04	-	-	Reactome
Collagen formation	88	13 (14.9)	8.21E-04	12 (13.8)	1.53E-03	Reactome
ARMS-mediated activation	16	5 (31.2)	1.26E-03	-	-	Reactome
Metabolism of nucleotides	81	12 (14.8)	1.39E-03	-	-	Reactome
RAF activation	5	3 (60.0)	1.49E-03	-	-	Reactome
Axon guidance	260	26 (10.1)	1.84E-03	-	-	Reactome
Signaling to RAS	25	6 (24.0)	1.87E-03	-	-	Reactome
Frs2-mediated activation	18	5 (27.8)	2.25E-03	-	-	Reactome
Hemostasis	472	40 (8.6)	2.98E-03	-	-	Reactome
Cell cycle	124	15 (12.1)	3.03E-03	-	-	KEGG
Peroxisome	81	11 (13.9)	3.58E-03	-	-	KEGG
FRS2-mediated cascade	39	7 (18.4)	4.00E-03	-	-	Reactome
MEK activation	7	3 (42.9)	4.80E-03	-	-	Reactome
Pentose phosphate pathway (hexose monophosphate shunt)	8	3 (42.9)	4.80E-03	-	-	Reactome
Proteoglycans in cancer	226	22 (9.8)	5.71E-03	-	-	KEGG
Integrin cell surface interactions	84	11 (13.1)	5.76E-03	-	-	Reactome
Regulation of actin cytoskeleton	215	21 (9.9)	6.23E-03	-	-	KEGG
PI3K-Akt signaling pathway	346	30 (8.7)	7.13E-03	30 (8.7)	7.13E-03	KEGG
Vitamin C (ascorbate) metabolism	8	3 (37.5)	7.37E-03	3 (37.5)	7.37E-03	Reactome
Serine biosynthesis	3	2 (66.7)	7.60E-03	2 (66.7)	7.60E-03	Reactome
Glyoxylate and dicarboxylate metabolism	24	5 (20.8)	8.52E-03	6 (25.0)	1.08E-03	KEGG
ERK1 activation	3	2 (66.7)	8.60E-03	2 (66.7)	8.60E-03	Reactome
PERK regulated gene expression	3	2 (66.7)	8.60E-03	2 (66.7)	8.60E-03	Reactome
Viral carcinogenesis	207	20 (9.7)	9.29E-03	20 (9.7)	9.29E-03	KEGG
Nuclear envelope breakdown	16	4 (25.0)	9.45E-03	4 (25.0)	9.45E-03	Reactome
Renin-angiotensin system	17	4 (23.5)	9.53E-03	4 (23.5)	9.53E-03	KEGG
Apoptotic cleavage of cellular proteins	40	-	-	10 (26.3)	1.48E-05	Reactome
Apoptosis	109	-	-	16 (15.1)	9.17E-05	Reactome
Complement and coagulation cascades	69	-	-	11 (16.2)	6.21E-04	KEGG
Complement cascade	79	-	-	11 (14.5)	1.60E-03	Reactome

KEGG, Kyoto Encyclopedia of Genes and Genomes; CCT/TriC, chaperonin containing t-complex polypeptide 1; MAP, mitogen-activated protein; TCA, tricarboxylic acid cycle; Frs2, fibroblast growth factor receptor substrate 2; PI3K, phosphoinositide 3-kinase; PERK, protein kinase RNA-like endoplasmic reticulum kinase; ERK, extracellular signal-regulated kinases.

**Table II:** Summary of secreted and membrane proteins in upregulated and downregulated human breast cancer proteins.

		Membrane		
		
	Secreted, n	Type I, n	Type II, n	Type III, n
Upregulated	137	33	94	115
Downregulated	126	50	122	166
